# Portal protein functions akin to a DNA-sensor that couples genome-packaging to icosahedral capsid maturation

**DOI:** 10.1038/ncomms14310

**Published:** 2017-01-30

**Authors:** Ravi K. Lokareddy, Rajeshwer S. Sankhala, Ankoor Roy, Pavel V. Afonine, Tina Motwani, Carolyn M. Teschke, Kristin N. Parent, Gino Cingolani

**Affiliations:** 1 Department of Biochemistry and Molecular Biology, Thomas Jefferson University, 233 South 10th Street, Philadelphia, Pennsylvania 19107, USA; 2 Department of Biochemistry and Molecular Biology, Rutgers University, 683 Hoes lane, Piscataway, New Jersey 08854, USA; 3 Molecular Biophysics & Integrated Bioimaging Division, Lawrence Berkeley National Laboratory, Berkeley, California 94720, USA; 4 Department of Molecular and Cell Biology Department of Chemistry, University of Connecticut, 91N. Eagleville Road, Storrs, Connecticut 06269, USA; 5 Department of Biochemistry and Molecular Biology, Michigan State University, East Lansing, Michigan 48824, USA; 6 Institute of Biomembranes and Bioenergetics, National Research Council, Via Amendola 165/A, 70126 Bari, Italy

## Abstract

Tailed bacteriophages and herpesviruses assemble infectious particles via an empty precursor capsid (or ‘procapsid') built by multiple copies of coat and scaffolding protein and by one dodecameric portal protein. Genome packaging triggers rearrangement of the coat protein and release of scaffolding protein, resulting in dramatic procapsid lattice expansion. Here, we provide structural evidence that the portal protein of the bacteriophage P22 exists in two distinct dodecameric conformations: an asymmetric assembly in the procapsid (PC-portal) that is competent for high affinity binding to the large terminase packaging protein, and a symmetric ring in the mature virion (MV-portal) that has negligible affinity for the packaging motor. Modelling studies indicate the structure of PC-portal is incompatible with DNA coaxially spooled around the portal vertex, suggesting that newly packaged DNA triggers the switch from PC- to MV-conformation. Thus, we propose the signal for termination of ‘Headful Packaging' is a DNA-dependent symmetrization of portal protein.

## Abstract (web summary)

Tailed bacteriophages assemble empty precursor capsids known as procapsids that are subsequently filled with viral DNA by a genome-packaging motor. Here the authors present a structure-based analysis that suggests the signal for termination of genome packaging is achieved through a DNA-dependent symmetrization of portal protein.

Tailed bacteriophages, nature's most abundant viruses, and herpesviruses assemble empty precursor capsids known as procapsids that are subsequently filled with viral DNA by a powerful, virus-encoded genome-packaging motor^1^,^2^. Procapsids are built by multiple copies of coat and scaffolding protein polymerized into an icosahedral shell that incorporates one dodecameric portal protein at a special 5-fold vertex. Portal protein forms a channel for bidirectional passage of viral DNA, which can move in and out of the virus head^3^,^4^ and provides an attachment point for the tail apparatus in tailed bacteriophages. In the procapsid of bacteriophage P22, the portal protein makes intimate contacts with coat and scaffolding proteins^5^,^6^, while in the mature virion, the portal assembly also interacts intimately with viral genome and tail components^7^,^8^. Structural studies on purified portal oligomers^9^,^10^,^11^,^12^,^13^,^14^,^15^,^16^ and *in situ* visualization of portal assemblies in virions^7^,^17^,^18^,^19^ revealed a conserved ‘portal-fold' that promotes assembly of large dodecameric assemblies (∼0.4–1 MDa) despite minimal sequence conservation.

Packaging of viral genomes inside procapsids is powered by an ATP-dependent motor that assembles at the portal vertex^20^,^21^. In P22, this motor consists of a large^22^ (L-) and small^23^ (S-) terminase subunit assembled in an oligomeric complex^24^. Encapsidation of DNA inside P22 procapsid proceeds by ‘Headful Packaging', a packaging strategy whereby the length of the DNA encapsulated inside the procapsid is determined by the interior volume of the mature phage particle^25^. DNA packaging causes refolding of P22 coat protein and release of the scaffolding protein, resulting in ∼10% expansion and angularization of the icosahedral capsid^26^. After ∼43 kilobase pairs of genome have been encapsulated and the capsid is full, the nuclease domain of L-terminase cleaves DNA, releasing P22 genome and causing dissociation of terminase. The portal is then sealed by tail factors gp4 (ref. 27), gp10 (ref. 28) and gp26 (refs 29, 30) that stabilize the genome inside P22 capsid.

In this paper, we have determined the quaternary structure of P22 portal protein that exists in procapsid. The atomic structure of this pre-packaging intermediate together with the structure of P22 portal in mature virion^13^ shed light on a dramatic and unexpected structural switch in the portal vertex that accompanies viral genome-packaging.

## Results

### A distinct conformation of P22 portal protein in procapsid

**Figure 1:**
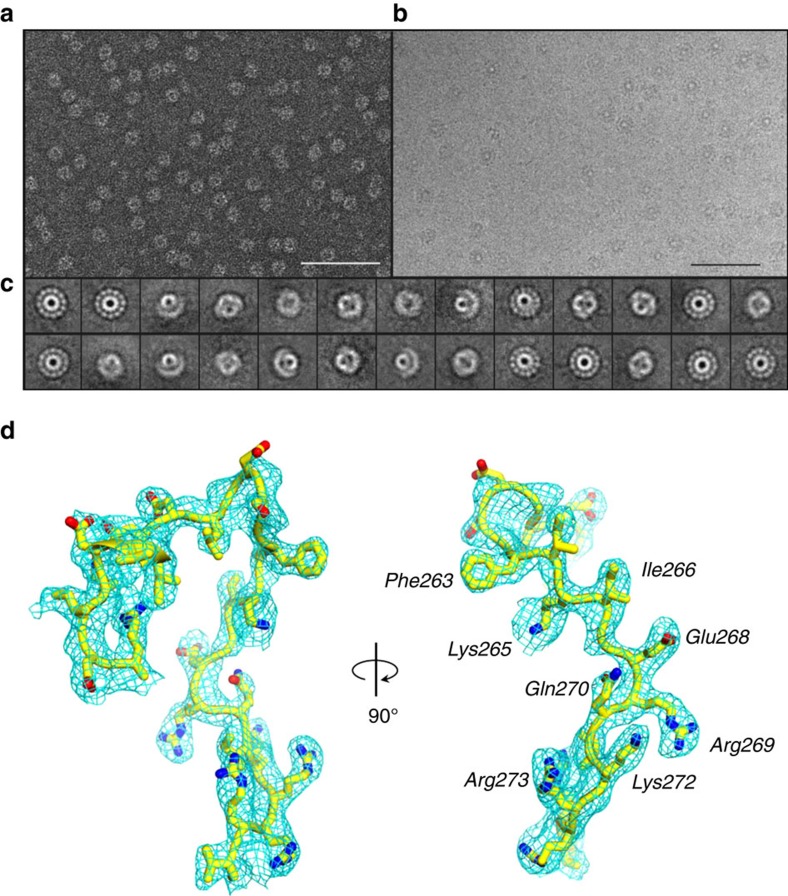
Evidence for a conformation of P22 portal protein by cryo-EM. (**a**) Representative micrograph of PC-portal negatively stained with 1% uranyl formate (Scale bar, 1 μm). (**b**) Representative micrograph of frozen-hydrated PC-portals (Scale bar, 1 μm). (**c**) Selected, reference-free 2D class averages of frozen-hydrated particles showing top and side projection views of PC-portal. (**d**) A σ_A_-weighted 2Fo–Fc difference electron density map computed at 3.30 Å resolution is displayed around a portion of the PC-portal protein model (*Arg249*–*Val276*), which is shown as sticks. The density is displayed as cyan mesh contoured at 1.65σ above background.

**Figure 2:**
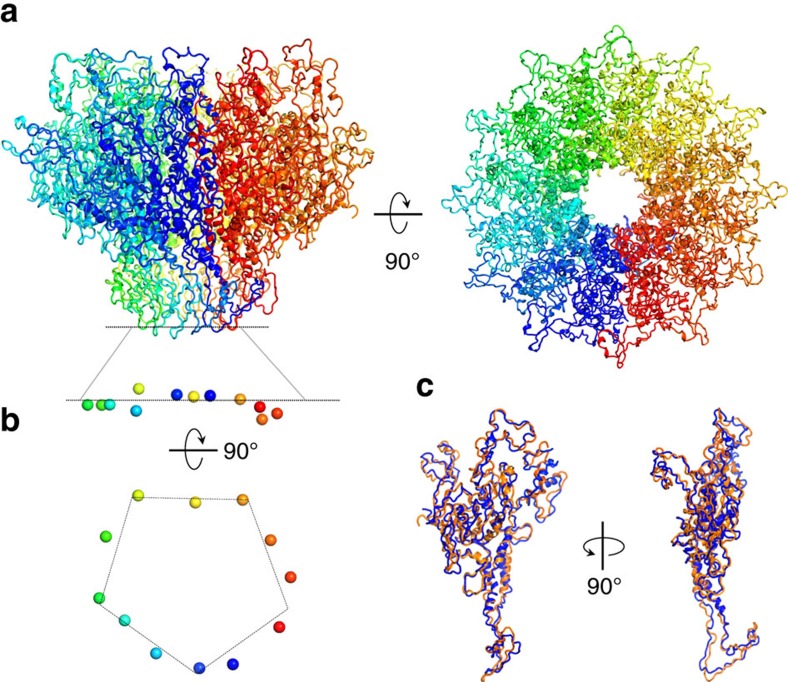
Crystal structure of PC-portal core at 3.30 Å resolution. (**a**) Ribbon diagram of the crystal structure of PC-portal protein core shown in side (left panel) and top (right panel) view. (**b**) Asymmetry in portal protein subunits. Side and bottom view of the side chain oxygen atom of Asn380 (shown as spheres) from each of the 12 subunits. (**c**) Superimposition of two most dissimilar subunits of PC-portal core, namely chain A (in blue) and chain J (in orange) (RMSD=3.4 Å).

X-ray structures of P22 portal protein core (portal-602) bound to gp4 (ref. 13) and of full length portal protein (portal-725) crystallized in the presence of *tert*-butanol were previously reported^31^. The portal core (res. 1–602) is identical in the two structures, while C-terminal residues 603–725 form a ∼200 Å helical ‘barrel'^8^ extending inside the virion. Docking inside the density map obtained from a cryo-electron microscopic (cryo-EM) asymmetric reconstruction of P22 mature virion^8^ revealed these X-ray models faithfully describe the conformation of portal protein in mature virion, when the capsid is filled with DNA and the portal is bound to gp4 (ref. 27). We will refer to this conformation as ‘Mature Virion-portal', or ‘MV-portal'. Surprisingly, the structure of MV-portal is significantly different from the density of portal protein in the 8.7 Å asymmetric reconstruction of P22 procapsid visualized *in situ*^5^, suggesting the existence of a procapsid-specific conformation of portal protein (referred to as ‘ProCapsid-portal' protein, or ‘PC-portal'). To validate this hypothesis, we developed a method to purify and assemble the putative PC-portal intermediate by omitting the heat-shock step proven important for purification of naïve MV-dodecamers^32^. EM analysis revealed this sample assembles mainly into dodecameric rings (Fig. 1). Unfortunately, the preferential particle orientation (>72% of all particles were found as ‘top' views) and the paucity of side views prevented high-resolution single particle analysis using cryo-EM. Nonetheless, we obtained small crystals of PC-portal protein core that yielded complete diffraction data to 3.30 Å resolution using a micro-focused X-ray beam (Table 1). Attempts to phase crystallographic intensities using the crystal structure of MV-portal protein core were unsuccessful, reflecting structural differences between the crystallized protein and our search model. Instead, a phasing model generated from the cryo-EM density of PC-portal protein was sufficient to obtain initial phases and build an atomic model that after several cycles of manual building was refined to an R_work/free_ of ∼29.5/31.5%, at 3.30 Å resolution (Table 1, Figs 1d and 2a). During refinement, it became apparent that PC-portal is profoundly asymmetric: the portal oligomer, which crystallizes in a tetragonal space group with a whole dodecamer in the asymmetric unit, could be refined only by gradually relaxing non-crystallographic symmetry (NCS) restraints that were omitted in the final stages of refinement. Imposing ‘strict' NCS (for example, NCS-constraints) destroys the model causing the R_free_ to raise to >50% (Supplementary Table 1). PC-portal is asymmetric in two respects: first, the twelve portal protomers adopt slightly different structures, with root-mean-square deviation (RMSD) of atomic positions ranging between 2.5 and 3.4 Å (average RMSD ∼2.85 Å) (Fig. 2b,c), strikingly higher than in MV-portal core (average RMSD ∼0.134 Å), refined at comparable resolution (∼3.25 Å) in complex with 12 copies of gp4 (ref. 13). Second, the 12 subunits of PC-portal occupy non-identical positions with respect to the 12-fold symmetry axis running along the centre of the oligomer. This asymmetry is particularly evident at the bottom of the DNA-channel, where the 12 subunits generated a *quasi*-5-fold surface (Fig. 2b).

**Figure 3:**
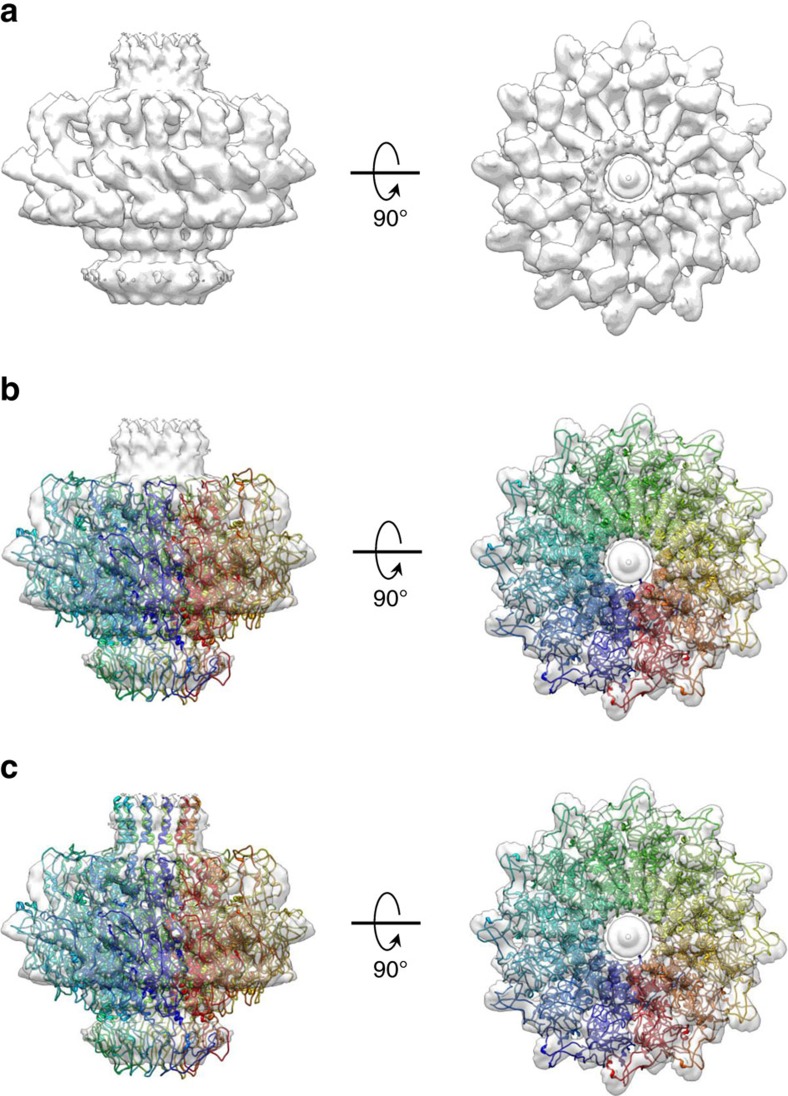
Architecture of the full length PC-portal protein. (**a**) 12-fold averaged cryo-EM map of portal protein (coloured in semi-transparent grey) extracted from the 8.7 Å asymmetric reconstruction of P22 procapsid (EMD-1828). (**b**) Crystal structure of PC-portal core overlaid to the cryo-EM map shown in (**a**). (**c**) A complete model of PC-portal protein that including the crystal structure of PC-portal protein core and modelled C-terminus spanning residues 602–631.

To validate the biological significance of the X-ray structure, the refined model was docked inside the EM-density of P22 PC-portal protein visualized *in situ*^5^ (Fig. 3a), revealing substantial agreement between the two structures. The fit is good, but not perfect (correlation coefficient, CC=0.74) (Fig. 3b), possibly owing to the fact the deposited *in situ* portal was 12-fold symmetrized^5^, eliminating the asymmetric features so prominent in the X-ray model. Additional density in the EM-map, not present in the crystallized construct that ends at residue 602, was modelled at the base of the barrel domain as ∼30 helical residues (Fig. 3c). The hybrid model of PC-portal protein obtained from X-ray crystallography and cryo-EM modelling, that includes residues 6–631, was rigid-body refined against the averaged cryo-EM density providing a substantially better fit (CC=0.83). By comparison, a model of MV-portal comprising an identical number of residues (res. 6–631) was also docked and rigid-body refined against the cryo-EM density yielding significantly lower correlation (CC=0.56), despite both MV-portal and cryo-EM density are perfectly symmetric. Thus, we have determined an atomic snapshot of a viral portal protein in its procapsid conformation.

### Structural changes accompanying portal protein maturation

**Figure 4:**
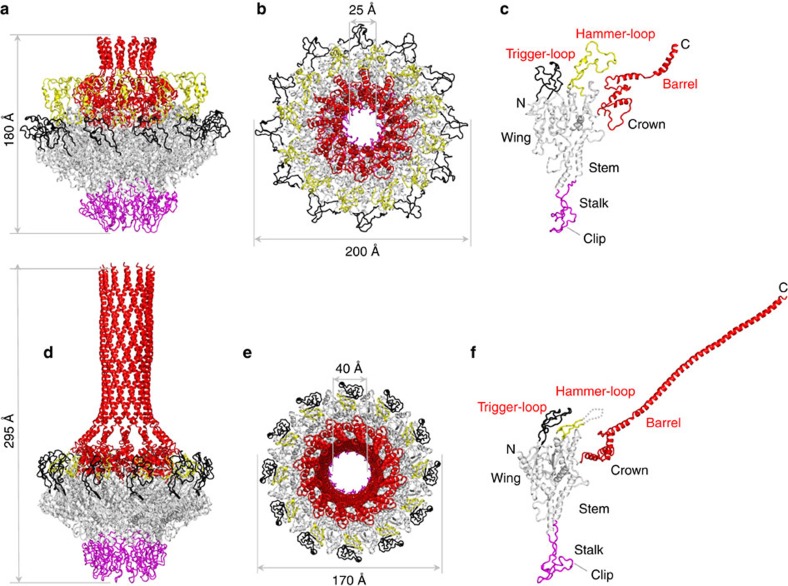
Structural comparison of PC- *versus* MV-portal protein. Ribbon diagram of P22 portal protein ring and protomer in procapsid (**a**–**c**) and mature virion (**d**–**f**) conformation. The portal oligomer is coloured in grey with stalk, trigger-loop, hammer-loop and crown-barrel coloured in magenta, black, yellow and red, respectively. The hammer-loop, invisible in MV-portal, is shown as dashes.

The structure of PC-portal protein (Fig. 4a–c) was compared with a complete atomic model of the full length MV-portal (Fig. 4d–f) that we refined to an R_work/free_ of 23.9/25.9, at 7.0 Å resolution (Table 1). The two conformations superimpose with an RMSD of 4.4 Å, underscoring profound structural differences and explaining why it was impossible to phase diffraction intensities by molecular replacement using MV-portal core as the search model. The PC-portal protein is less compact, having a noticeable increase in external diameter from 170 to ∼200 Å. Despite an increase in outer dimension, the diameter of the DNA channel decreases in the PC-portal from ∼40 Å to ∼25 Å (Fig. 4b,e), which is close to the diameter of hydrated double stranded DNA (dsDNA)^23^. Residues lining the channel are therefore more likely to make direct contact with DNA during packaging in PC-portal, possibly explaining why certain mutations in portal protein affect the amount of DNA packaged in the procapsid^33^. The PC-portal has greater compression of stem helices that make an angle of 20° relative to the 12-fold portal axis as compared with 30° in the MV-conformation. This change in the angle of the stem helices with respect to this axis is responsible for the greater degree of compression in PC-portal. Finally, ∼100 residues in the barrel domain (res. 631–725) are not folded in PC-portal, in stark contrast to the MV-conformation where the C-terminus forms a 200 Å long barrel, tightly surrounded by viral DNA^8^,^13^.

To rationalize the conformational plasticity of P22 portal protein in the two conformational states, we analysed the structure of the portal protomer that consists of five regions (Fig. 4c,f): a central ‘wing', consisting of a flat domain of α/β-fold; a ‘stem' formed by two antiparallel α-helices making up most of the DNA channel; a ‘stalk' (res. 344–398) that forms the bottom of the portal ring and binds L-terminase and gp4 (ref. 13); a helical ‘crown' (res. 525–600) flexibly connected to the wing that extends into a helical ‘barrel' (res. 600–725), conserved in many *Podoviridae*^8^. In PC-portal, the wing has two surface exposed-loops that we named ‘trigger' (residues 226–277) and ‘hammer' (res. 456–505) (Fig. 4c). The trigger faces outwards toward the portal perimeter, while the hammer folds onto the portal surface making contacts with the crown. The hammer:crown interaction pushes the crown inside the DNA channel narrowing the lumen of the portal channel to <25 Å. This, in turn, forces the barrel α-helices to lose inter-helical contacts and unfold after residue 631 (ref. 5), explaining why only a small region of the barrel is visible in procapsid. In contrast, in MV-portal (Fig. 4f), the hammer-loop is disordered (that is, this loop is invisible in all crystal structures of MV-portal protein core 8,13), and the crown rotates outwards, enlarging the DNA channel and allowing barrel helixes to straighten up and make inter-helical contacts. Finally, as previously noticed^8^, the barrel helices are significantly more constricted in the presence of DNA than in the crystal structure.

### The barrel is mostly unfolded in the absence of DNA

**Figure 5:**
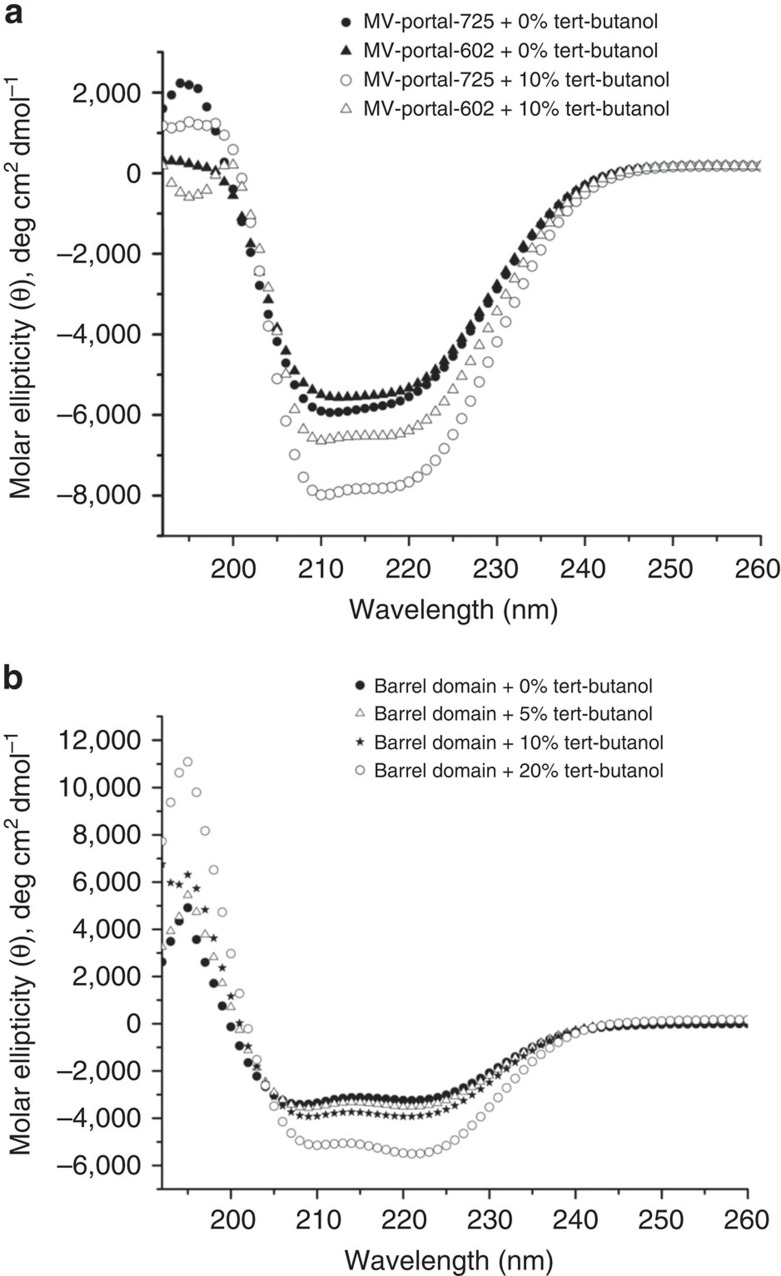
The barrel domain is unfolded in solution. (**a**) The far-UV CD spectra of portal-725 (black circle) and portal-602 (black triangle) dissolved at 1 μM final concentration, in 10 mM HEPES, pH 7.4 and 70 mM NaCl. Significant effect of 10% *tert*-butanol was observed for portal-725 (open black circle) as compared with portal-602 (open black triangle). (**b**) The far-UV CD spectra of the isolated barrel domain dissolved at 5 μM final concentration in 10 mM HEPES, pH 7.4 and 70 mM NaCl in presence and absence of *tert*-butanol. For each sample, CD spectra were measured at 10 °C in the presence of 0% (black circle), 5% (open black triangle), 10% (black star) and 20% (open black circle) *tert*-butanol.

Previous studies indicated the portal barrel is highly dynamic^34^ and susceptible to proteolysis^31^ in solution. To determine if the barrel is stably folded in the absence of viral DNA, we compared circular dichroism (CD) spectra of MV-portal-725 and MV-portal-602 (Fig. 5a). Secondary structure content estimated from the measured ellipticity using the K2d method^35^ revealed the two portal constructs contain ∼55% α-helical content and roughly equal amount (∼20–22%) of β-sheets and random coil conformations. If the barrel adopted the helical structure seen in crystal in solution (Fig. 4d), the total expected helical content would be 35% greater in MV-portal-725 than in MV-portal-602, which was not observed experimentally. Thus, the barrel is unfolded in solution, as seen in the cryo-EM reconstruction of P22 procapsid^5^. Addition of 10% *tert*-butanol, the kosmotropic (order-making) agent used for crystallization of MV-portal-725 (ref. 31), increased helicity by 30% (Fig. 5a), pointing to an inducible nature of the helical barrel. Likewise, the isolated barrel (res. 602–725) was partially helical in solution, but acquired significant helical content in the presence of tert-butanol (Fig. 5b), yet remaining monomeric, as assessed by gel filtration chromatography (Supplementary Fig. 1). This suggests the isolated barrel helices form weak interactions, not sufficient to confer stable folding to the barrel, although a weak property to adopt a coiled-coil conformation can be identified from the amino acid sequence using conventional bioinformatics software^8^. We propose that *in vivo* the barrel straightens when the helices are forced laterally by packaged DNA (and possibly by the presence of ejection proteins), which can be mimicked *in vitro* by adding ∼10% *tert*-butanol. This observation may help reconcile why the barrel is visible in the reconstruction of P22 mature virion that is filled with DNA^7^,^8^ but is invisible both in the procapsid^5^ and in the tail complex isolated from infectious virions^36^.

### Structural determinants for binding to large terminase

**Figure 6:**
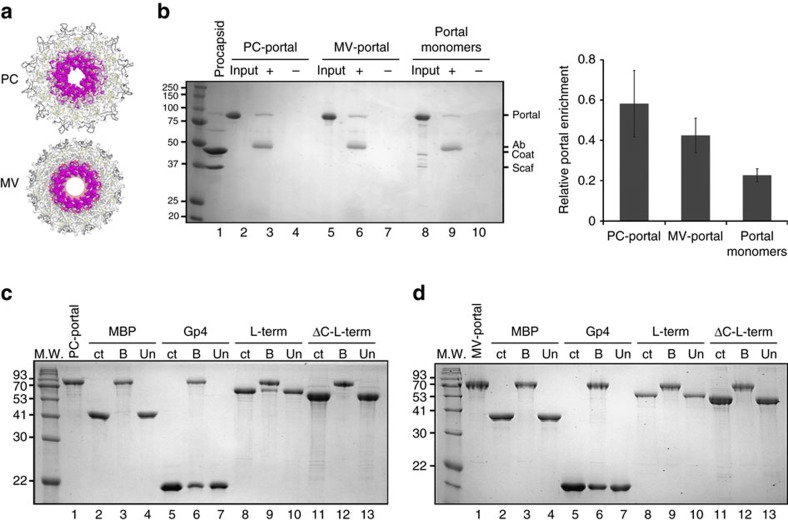
Large terminase binds exclusively to PC-portal. (**a**) Bottom views of PC-portal (top) and MV-portal (bottom) with residues 375–385 in the stalk loop shown as spheres. (**b**) Portal protein immunoprecipitation assay. Left panel: Coomassie blue stained SDS–PAGE of a representative portal protein immunoprecipitation. ‘Input': 2.5 μg of portal proteins; ‘+': proteins immunoprecipitated by the anti-stalk antibody; ‘−': proteins incubated with Protein A agarose beads without the antibody. The migration of portal protein, antibody IgG band (Ab), coat protein and scaffolding protein (Scaf) is indicated. Right panel: quantification of portal protein band relative to the antibody band for three experiments, as quantified by densitometry (the error bar represents standard deviation). (**c**,**d**) SDS–PAGE analysis of L-terminase binding to PC- (**c**) and MV-portal protein (**d**) immobilized on CNBr beads. Portal-beads (lane 1) were used to selectively pull-down MBP (lane 3), gp4 (lane 6), L-terminase (lane 9), ΔC-L-terminase (lane 12). ‘ct': control, ‘B' and ‘Un' are fractions bound and unbound to portal beads.

The clip region of portal protein stalk recruits L-terminase to promote genome-pumping^37^. In PC-portal this region is flat and flowered out exposing a large surface area (Fig. 4c), remarkably different from the narrower conformation seen in MV-portal bound to gp4 (Fig. 4f)^13^. To study the structural plasticity of this critical region of portal protein, we generated an anti-peptide antibody to residues 375–385 in the stalk loop of P22 portal protein (Fig. 6a). We used this antibody in immunoprecipitation (IP) experiments to assess the conformation of the loop in the different states of the portal assembly. We found the anti-stalk antibody was best able to interact with PC-portal rings in solution (Fig. 6b, lane 3), with slightly reduced interaction with MV-portal (Fig. 6b, lane 6). In contrast, we observed negligible interaction with portal monomers (Fig. 6b, lane 9), indicating a change in the stalk conformation that occurs during assembly. In addition, the portal protein in genuine PC (2^−^13^−^ phage infection), in empty matured heads (4^−^13^−^ infection), or tail-less phages (9^−^13^−^ infection) (Supplementary Fig. 2) did not interact with the antibody (no phage proteins seen in the+antibody lanes), suggesting there are additional conformational changes that can occur in portal protein upon assembly with coat and scaffolding protein. Logically, the stalk of portal protein must be mobile as it is able to interact with the terminase protein in the PC form and is released when headful packaging is completed.

To determine if the conformation of portal protein affects the binding affinity for the packaging L-terminase subunit, we coupled purified PC- and MV-portal rings to agarose beads and assayed portal-beads for binding to P22 L-terminase pre-complexed with non-hydrolyzable ATP. As expected Maltose Binding Protein (MBP), used herein as a negative control, failed to associate with either conformation of portal protein (Fig. 6c,d lane 3), while the tail factor gp4 (ref. 27) bound both conformations of portal protein (Fig. 6c,d lane 6). Quantification of gp4 bound to MV- and PC-portal beads revealed a statistically equal number of gp4 equivalents (11±1 versus 11.4±1), consistent with the stoichiometry observed biochemically^27^, crystallographically^13^ and by cryo-EM^8^,^15^,^38^. Gp4 binds like a ribbon to the lateral bottom surface of portal, inserting an extended C-terminal tail at the protomer:protomer interface^13^,^32^ (Supplementary Fig. 3a–d). Unlike gp4, L-terminase is thought to associate directly with the outer lumen region of portal protein^37^,^39^,^40^ that undergoes dramatic conformational changes both in tertiary structure of the clip and quaternary structure of the channel, which is *quasi*-5-fold symmetric in PC-portal (Fig. 2b) and perfectly 6-fold symmetric in MV-portal (Fig. 6a). By pull-down assay, L-terminase associated with PC-portal protein (Fig. 6c, lane 9) but not with MV-portal (Fig. 6d, lane 9), suggesting only the procapsid intermediate of portal is competent for DNA-packaging. A mutant of L-terminase lacking C-terminal residues 483–499 (ΔC-L-terminase)^24^ was, however, unable to associate with PC-portal protein (Fig. 6c,d lane 12), suggesting the C-terminal tail of P22 L-terminase contains a binding site for portal protein, as previously reported for phage T3 (ref. 41) and λ (ref. 42). A stretch of basic residues and prolines in L-terminase conserved in all P22-like bacteriophages (480-IRKPKEKKIPAPIRPVRR-497) is likely involved in this interaction. Thus, P22 portal protein switches from a high affinity state for L-terminase in procapsid, to a symmetric conformation in mature virion that has negligible affinity for the packaging L-terminase subunit.

### PC-portal structure is incompatible with packaged DNA

**Figure 7:**
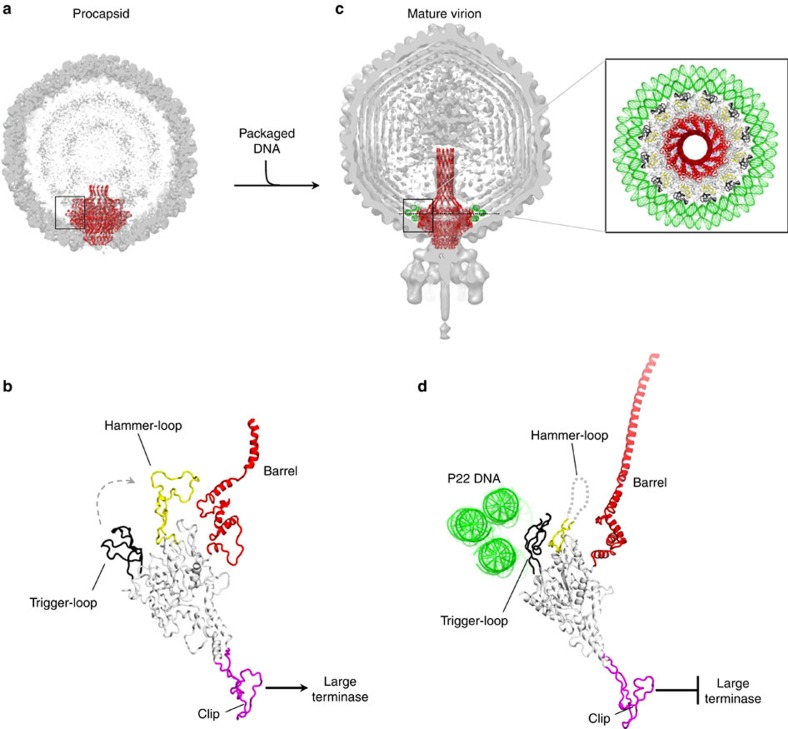
Modelling portal protein maturation. (**a**) Cut-open representation of P22 procapsid (EMD-1827) with the ribbon structure of portal protein (in red) overlaid to the cryo-EM density. (**b**) Magnified side view of the PC-portal protomer found in procapsid. (**c**) Cut-open representation of P22 mature virion (EMD-1220) with the ribbon structure of portal protein (in red) overlaid to the cryo-EM density. Magnified on the right is a top view of MV-portal protein surrounded by three rings of DNA visible in the cryo-EM reconstruction. (**d**) Magnified side view of the MV-portal protomer surrounded by the three rings of DNA.

The X-ray structures of PC-portal core and MV-portal protein, together with previous asymmetric cryo-EM reconstructions of P22 mature virion^8^ and procapsid^5^ provide a detailed description of the initial and final states of the portal vertex during assembly. We used molecular modelling to rationalize the dynamics of portal protein maturation. At first, we docked the X-ray structure of PC-portal into the asymmetric cryo-EM reconstruction from empty procapsids (Fig. 7a). Although the asymmetrized density of portal protein is significantly weaker than the deposited symmetrized map (Fig. 3a), the structure of PC-portal can be placed manually, or computationally (using a six-dimensional search with phases), inside the EM-reconstruction. In PC-portal protein, the trigger-loop is locked in an open conformation (Fig. 7b), possibly by making contacts with scaffolding protein^5^, while the hammer-loop rests folded preventing barrel extension and narrowing the DNA-binding channel to >25 Å. On the opposite side, the intrinsic asymmetry of PC-portal provides a plastic binding surface for L-terminase that in many phages assembles into a pentamer^14^,^37^,^40^,^43^. As DNA begins to fill the procapsid, DNA spools coaxially around the perimeter of the portal wing (Fig. 7c), as seen in the cryo-EM reconstructions of P22 (ref. 7), T7 (ref. 44) and epsilon 15 (ref. 19). Three rings of DNA surrounding the perimeter of MV-portal protein have strong density in the reconstruction of P22 mature virion (enlarged panel in Fig. 7c) and their shape is complementary to the trigger loop of MV-portal, which faces inwards toward the hammer-loop (Fig. 7d). Modelling these DNA rings around the structure of PC-portal, which is 30 Å wider than MV-portal (200 versus 170 Å), reveals the position of the trigger-loop in PC-portal is incompatible with DNA spooling around portal protein, as severe clashes would occur between ∼45 residues in the trigger-loop (res. 275–230) and the bottom DNA ring (Supplementary Fig. 4). As previously hypothesized^7^, it is possible that the tension generated by DNA spooling inside procapsid and tightening around the portal, which builds up during packaging^45^, forces the trigger-loop to swing by 90° clockwise, destabilizing the hammer-loop that then becomes unfolded. The lost grip of the hammer-loop on the crown may allow this domain to rotate, straightening the barrel helices that find side-by-side complementarity and become folded, concomitant with DNA filling the capsid (Fig. 7d). The outside surface exposed by the barrel helices is mildly acidic (Supplementary Fig. 5), suggesting the barrel straightens during DNA packaging as a result of the repulsion with negatively charged DNA, as opposed to a stabilization induced by contacts with DNA. While the portal shrinks in diameter from ∼200 to 170 Å and the barrel folds, a long-range conformational change is propagated from the crown to the stalk through the entire structure of portal channel, leading to symmetrization and loss of binding affinity for L-terminase (Fig. 7d). This may allow the terminase's nuclease domain to cleave dsDNA, marking the end of genome packaging. Because L-terminase dissociates from the portal vertex only at the end of DNA-packaging, which lasts in the order of minutes, it is possible the complete conversion from a high affinity (PC-portal) to a low affinity (MV-portal) conformation for terminase occurs slowly and intermediate states of PC-portal are populated, as also suggested by our anti-stalk Ab.

## Discussion

In this paper, we describe an immature conformation of the portal protein found in the P22 procapsid, before genome packaging, that we named PC-portal. This intermediate has eluded cryo-EM studies for over a decade^7^,^15^,^36^, possibly because of the inherent challenge of distinguishing PC-portal from MV-portal rings in 2D-projections, using single particle reconstruction methods. Crystallization was successful in isolating PC-portal from a complex mixture of portal rings and monomers. We solved a crystal structure of this intermediate at 3.30 Å resolution and validated this structure in the asymmetric cryo-EM reconstruction of P22 procapsids. Unlike MV-portal, PC-portal is strikingly asymmetric and exposes a *quasi*-5-fold symmetric surface to the packaging L-terminase subunit. We validated this observation biochemically and found that the portal protein switches from a high affinity state for L-terminase in procapsid, to a symmetric conformation in the mature virion that has negligible affinity for the packaging motor. Modelling studies using the X-ray structures of PC-portal core and MV-portal protein described in this paper, together with previous asymmetric cryo-EM reconstructions of P22 mature virions^8^ and procapsids^5^ reveal the quaternary structure of PC-portal is incompatible with packaged DNA coaxially spooled around the portal vertex, lending support to a model whereby newly packaged DNA triggers the conformational switch from PC- to MV-portal protein. Our findings have several implications important to understanding the role of portal protein in genome-packaging and icosahedral capsid maturation.

*First*. The majority of DNA viruses on earth assemble empty procapsids from three proteins: coat, scaffolding and portal protein. It is well established that coat and scaffolding proteins undergo large conformational changes during capsid assembly and maturation^26^. Our work adds a missing piece to this puzzle, providing structural evidence that the portal vertex is also sensing assembly by undergoing DNA-induced quaternary structure maturation. The structural conservation of the portal protein fold^14^, even in viruses of different lineages, makes it likely the PC- to MV-structural maturation described in this paper represents a general principle of virus morphogenesis, conceptually similar to the quaternary structure conformational changes in coat and scaffolding proteins occurring upon genome-packaging. Although the exact time-scale of this maturation is unknown, we speculate the switch from PC- to MV-conformation occurs concomitantly with icosahedral capsid expansion and proceeds through short-lived intermediates. As also suggested by our antibody IP experiment, portal protein loops exposed to L-terminase and DNA are highly plastic and so is the portal barrel, which is unstructured in the absence of DNA but becomes stabilized at the end of packaging, surrounded by DNA. Thus, we propose the portal assembly samples intermediate conformations between PC- and MV-portal, which should be thought as crystallographic snapshots of the initial and final states of maturation, informative but by no means descriptive of all conformational states this protein samples during structural maturation. We expect our findings will spur renewed interest in studying the conformational dynamics and structural maturation of the portal vertex in other bacteriophages and herpesviruses, leading to a better understanding of icosahedral capsid assembly and maturation.

*Second*. The finding that portal protein switches from an asymmetric PC-conformation with high affinity for L-terminase to a symmetric MV dodecamer with negligible affinity for the packaging motor provides a reading frame to decipher the cascade of events accompanying headful packaging, one of nature's most common strategies to package genomes into empty procapsids. We propose that a DNA-induced symmetrization of portal protein during genome packaging represents the signal that switches the immature, intrinsically asymmetric PC-portal into a symmetric oligomer (MV-portal) resulting in loss of binding affinity for L-terminase. This decrease in affinity could slow down translocation of DNA and in turn enhance the nuclease activity of L-terminase by removing the inhibitory effect of portal protein^22^. Other factors like S-terminase and the tail factor gp4 are also likely to play a role in this complex and concerted reaction. For instance, S-terminase is known to inhibit the nuclease activity of L-terminase^22^,^46^ possibly prompting L-terminase to cleave DNA, and terminating the packaging reaction. Likewise, gp4 can bind either PC- and MV-conformation and may use its flexible structure to remain bound to PC-portal while the assembly undergoes (or simply terminates) maturation, also contributing to displace L-terminase and activating L-terminase nuclease activity by removing the inhibitory effect of portal protein^22^. Thus, a structural rearrangement in the portal vertex could be one of the ways the portal vertex senses the internal pressure and signals the motor to terminate genome-packaging.

*Third*. The discovery of a *quasi*-5-fold asymmetric conformation of portal in procapsid resolves a conundrum in structural virology possibly explaining the ‘symmetry mismatch' between the dodecameric portal vertex and the 5-fold symmetric terminase. In many phages (for example, P22 (ref. 22), Sf6 (ref. 47), SPP1 (ref. 48) and T4 (ref. 49), P74-26 (ref. 50)), the L-terminase subunit is monomeric in solution but forms a pentamer on binding to the procapsid, which exposes an apparently 12-fold symmetric portal vertex. We propose that by adopting an asymmetric conformation, PC-portal provides a structural template for L-terminase to oligomerize upon binding into a functional pentamer, as seen in T4 (refs 14, 37), T7 (ref. 40) and Phi29 (ref. 43). Furthermore, the finding of an asymmetric portal vertex in procapsid resonates well with recent single molecule studies of packaging motors and provides a reading frame to decipher the functional asymmetry observed during the burst phase of the packaging cycle^45^. For instance, asymmetry in the portal vertex could be transferred to the terminase oligomer to generate non-equivalent structural environments in the ATPase active site of different L-terminase protomers. Asymmetry is indeed central to the catalytic mechanisms of multi-subunit, rotary enzymes such as the F_o_F_1_-ATP synthase^51^ and is likely to play an important role in viral packaging motors as well.

*Fourth*. Global^3^,^9^,^52^ or local^11^ conformational changes in the portal vertex accompanying DNA packaging have been postulated in several models put forward in recent years to explain the process of mechano-chemical force generation in viral genome packaging motors. Our discovery of a built-in plasticity in P22 portal vertex that exists in at least two conformational states (for example, PC- and MV-conformation) provides new experimental ground to study the involvement of portal protein in genome packaging. Indirect evidence for a conformational plasticity of P22 portal protein was provided over two decades ago by the identification of gain-of-function point mutations in portal that result in encapsidation of ∼2,000 extra base pairs of DNA during packaging^33^. Further studies are needed to assess if these mutations stabilize the conformation of PC-portal, which has high-affinity for L-terminase or, conversely, destabilize MV-portal, slowing down motor dis-attachment and enabling genome packaging to endure longer. Finally, the narrower diameter of the DNA channel of PC- versus MV-portal observed in our X-ray structures provides indirect evidence in support of the ‘*push through a one-way valve*' model^53^, which predicts the portal protein channel has a ‘DNA-retention' function^54^ serving as a one-way valve preventing backward motion of DNA during packaging.

*Fifth*. The unexpected finding of an asymmetric conformation of PC-portal suggests that caution should be exercised when imposing symmetry during structural analysis of portal oligomers, particularly in single-particle methods that greatly rely on density averaging to obtain high resolution 3D-reconstructions. This problem also exists, but is perhaps less significant, in X-ray crystallography, whereby the *free* R provides an excellent indicator of ‘genuine' symmetry during refinement (Supplementary Table 1). Future efforts should be devoted to solving high-resolution asymmetric reconstructions of portal assemblies bound to terminase subunits that capture, and faithfully preserve, the built-in asymmetry in PC-portal, which, we anticipate, is transferred to the packaging motor during DNA pumping.

In conclusion, our data not only support the hypothesis that P22 portal protein functions like a DNA-sensor that measures the amount of DNA packaged inside the head and signals for termination of packaging^7^,^33^, but also reveal specific structural determinants that allow PC-portal to sense the amount of genome packaged inside the capsid and, lastly, by adopting a symmetric quaternary structure, send a termination signal to L-terminase to end genome-packaging. Thus, though the portal protein does not rotate during DNA-packaging^55^,^56^, in this paper we show it undergoes a dramatic conformational change, switching from a PC-conformation poised for high affinity binding to L-terminase to a symmetric, highly folded structure in mature virion that has low affinity for the packaging motor and is stabilized by attachment of tail factors.

## Methods

### Biochemical methods

P22 portal protein core (res. 1–602, or portal-602) and full length portal protein (res. 1–725, or portal-725) were cloned in pET21b and expressed in BL21 (DE3) *E. coli* cells. After growth at 37 °C to an *A*_600_ of 0.6, expression was induced with 1 mM IPTG and shaking for 4–5 h. To oligomerize and purify PC-portal, both protein-725 and portal-602, cells were collected and lysed by sonication in Lysis buffer (300 mM NaCl, 20 mM Tris–HCl pH 8.0, 30 mM imidazole, 5 mM β-mercaptoethanol, 1 mM PMSF) and C-terminal 6 × -His tagged portal-602 was purified by metal-chelating affinity chromatography using High Affinity Ni-NTA Resin (GenScript). The beads were extensively washed with Lysis buffer and with final 50 m wash steps also containing 40 and 50 mM imidazole. The protein was eluted with Elution buffer (50 mM NaCl, 20 mM Tris–HCl pH 8.0, 500 mM imidazole, 5 mM β-mercaptoethanol, 1 mM PMSF) and dialysed overnight in 5 l of Dialysis buffer (25 mM NaCl, 20 mM Tris–HCl pH 8.0, 0.5 mM EDTA, 5 mM β-mercaptoethanol, 0.1 mM PMSF) to remove imidazole. Portal-602 was then concentrated to 100–200 mg ml^−1^ and incubated at room temperature for 24 h to promote oligomerization. The protein was then purified by gel filtration chromatography on a Superose 6 column (GE Healthcare) equilibrated with Dialysis buffer. MV-portal protein was purified by metal affinity chromatography with Ni-agarose beads followed by gel filtration chromatography on a Superose 12 column (GE Healthcare)^32^. Recombinant L- and S-terminase subunits were expressed in BL21 (DE3) *E. coli* cells and purified by affinity chromatography on either Ni-agarose or amylose beads, followed by gel filtration chromatography using a HiLoad 16/600 Superdex 200 pre (GE Healthcare)^22^,^23^. Truncated ΔC-L-terminase (res. 1–482) was generated by introducing a stop codon at position 483 and expressed and purified like full length L-terminase^23^. Gp4 was expressed in BL21 (DE3) *E. coli* cells and purified by metal affinity chromatography with Ni-agarose beads followed by gel filtration chromatography on a Superdex 75 (GE Healthcare)^13^. Binding of purified L-terminase to PC-portal and MV-portal immobilized on CNBr-activated Sepharose 4B (GE Healthcare) was carried out as described in our previous publication^57^.

### Immunoprecipitation

A polyclonal antibody was generated (Pacific Immunology Corp.) against portal protein residues 375–385 (Cys-NRTDENSGDLP) present in the stalk loop of P22 portal protein. For immunoprecipitation, of 5 μg of the portal proteins (PC-portal, MV-portal, and portal monomers) and 10 μg of the control samples (WT 2^−^13^−^ PC, pPC, 4^−^13^−^ heads, and 9^−^13^−^ phage) were used. The procapsids assembled *in vivo* but without the portal (using the plasmid pPC) and the genuine PC (2^−^13^−^ phage infection), empty matured heads (4^−^13^−^ infection), tail-less phages (9^−^13^−^ infection) were prepared as reported in a previous publication^58^. The 9^−^13^−^ tail-less phages were further purified by caesium chloride density gradient centrifugation^59^. Each of the samples was incubated with 8 μg anti-stalk peptide antibody in 250 μl Buffer B (25 mM Tris, 50 mM NaCl, 2 mM EDTA, pH 7.6)+0.1 Triton-X 100. To keep the DNA containing the 9^−^13^−^ phage from breaking, this sample was incubated in 250 μl TM buffer (10 mM Tris, 100 mM MgCl_2_, pH 7.5)+0.1% Triton-X 100. To each sample, 40 μl of 1:1 slurry of immobilized Protein A Agarose beads (Pierce) was added and incubated for 2 h at 4 °C on. After incubation, the immune complexes bound to the beads were collected by centrifugation at 13,200 r.p.m. at 4 °C for 30 s using a microfuge. The beads were washed two times with 500 μl of Buffer B+0.1% Triton-X 100 or TM buffer+0.1% Triton-X 100 and two times with 500 μl of Buffer B or TM buffer. Finally, the portal protein complex was eluted from the beads by boiling at 95 °C in reducing sample buffer (20 mM Tris pH 7.6, 0.1% SDS, 20% glycerol, 1 mM EDTA and 1.5 ml β-mercaptoethanol) for 5 min and separated on a 12.5% SDS–polyacrylamide gel (SDS–PAGE). The control samples were separated on 10% SDS–PAGE.

### Electron microscopy

Small (3.5 μl) aliquots of purified full length PC-portal (∼1.2 μM) were applied to holey Quantifoil grids that had been plasma cleaned for 20 s in a Fishione model 1,020 plasma cleaner. Grids were then blotted with Whatman filter paper for ∼5 s, plunged into liquid ethane, and transferred into a precooled, Gatan 914 holder, which maintained the specimen at liquid nitrogen temperature. Micrographs were recorded using a Direct Electron DE-20 camera (Direct Electron, LP, San Diego CA), in an JEOL 2200FS microscope operated at 200 keV, using low-dose conditions controlled by SerialEM^60^. Images were collected at a nominal magnification of × 25,000 (∼2.3 Å pixel^−1^), using an energy filter set to a 35 eV slit width, and objective lens settings ranging 1.9 to 3.0 μm underfocus. The DE-20 camera was operated with 25 frames per second capture rate for a total exposure of 39 frames, and 40 e^−^ (Å^2^)^−1^ total dose. Movie correction and damage compensation was performed on whole frames using the Direct Electron software package, v2.8.1 (ref. 61). EMAN2 was used to extract individual particles and perform 2D reference-free class averages. In total, 5,919 particle images were extracted from 73 micrographs.

### Crystallographic methods

PC-portal protein core concentrated to 10 mg ml^−1^ was crystallized in the presence of 5% PEG 8,000, 10 mM Caesium Chloride at pH 5.6 at 22 °C. Plate-like crystals appeared after ∼3 months and were very fragile. All crystals were cryoprotected in mother liquor supplemented with 27% ethylene glycol and X-ray diffraction data were collected at 21-ID-F (LS-CAT) beamline at the Advanced Photon Source (APS). The best crystals diffracted to ∼3.3 Å resolution and were used to collect a complete and highly redundant dataset including 9,911,319 reflections. Diffraction spots were integrated and scaled with HKL2000 (ref. 62) and with the exception of overloads, no outliers were rejected during scaling resulting in a higher than usual *R*_sym_ ∼30.1 (though the Precision-indicating merging *R*-value, *R*_pim_ is ∼10.5) (Table 1). All crystals suffered from merohedral twinning with twinning fractions between 0.35–0.49 as revealed by *phenix.xtriage*^63^. Attempts to phase diffraction intensities by molecular replacement with *phaser*^64^ using the MV-portal core (pdb 3LJ5) as search model were unsuccessful. A search model was then generated using cryo-EM modelling. Briefly, we manually placed MV-portal-602 (pdb id 3LJ4) inside the asymmetric cryo-EM density of P22 procapsid (EMD-1828)^5^ and subjected it to iterative rounds of manual rebuilding in COOT^65^ and real space refinement with *phenix.real_space_refine*^66^. From this quasi-atomic model, the structure of one portal protomer was then extracted, idealized to improve stereochemistry and used to generate a homo-dodecameric ring inside the symmetrized 8.7 Å cryo-EM map of P22 PC-portal protein (EMD-1828) using MultiFit^67^. This 12-fold symmetric oligomer was then used as search model for molecular replacement as implemented in *phaser*^64^, which readily located an unambiguous solution (LLG >3,000). A complete model was obtained by alternating ∼20 rounds of manual model building in COOT^65^ with real space refinement in *phenix.real_space_refine*^66^ and positional and isotropic B-factor refinement in *phenix.refine*^63^ using torsion-angle NCS-restraints. When the *R*_free_ dropped below ∼35% Ramachandran, C-beta, secondary structure and rotamer restraints together with automatic weight optimization were also implemented in *phenix.refine*^63^ without imposing NCS restraints, that were found to severely distort the model geometry (Supplementary Table 1). Riding hydrogen atoms were also added to the model and used in the final stages of refinement to improve the overall geometry. Because of the merohedral twinning, only the first step of crystallographic refinement was carried out with a twin target function and twin law (−h, k, −l) using structural factor amplitudes with Fobs/σ_Fobs_>2.5 (for example, the total number of reflections available for refinement after imposing this cut-off is 173,371, corresponding to a completeness in resolution range of 85.3%). Detwinned, bulk-solvent corrected structure factor amplitudes were then used in all subsequent steps of refinement using a Maximum Likelihood target function in *phenix.refine*^63^. The final model has an *R*_work_/*R*_free_∼29.5/31.5%, calculated using reflections between 15 and 3.30 Å resolution (Table 1) (reflections used for *R*_free_ calculation were selected in 20 thin resolution shells). The final model has good geometry (RMSD_bond_=0.004 Å, RMSD_angle_=1.033°), and the Ramachandran plot shows 76.9% of residues in the most favored regions, 21.5% of residues in allowed regions, and only 1.4% of residues in disallowed regions, with no rotamer and C-beta outliers (Supplementary Table 1). Crystals of the full length MV-portal protein were obtained as described^13^,^31^. Complete diffraction data to ∼7.0 Å resolution were collected at beamline 14–1 at the Stanford Synchrotron Radiation Lightsource (SSRL). The structure of MV-portal-725 (pdb id 3LJ5) was refined against the new dataset using rigid body refinement, real space refinement and B-grouped refinement including 20 TLS groups using NCS-constraints, as implemented in *phenix.refine*^63^. The final portal-725 has *R*_work_/*R*_free_∼23.9/25.9%, using reflections between 15 and 7.0 Å resolution (Table 1) (reflections used for *R*_free_ were selected in 20 thin resolution shells). The final model has considerably better geometry than the model previously deposited (RMSD_bond_=0.004 Å, RMSD_angle_=1.040°) and the Ramachandran plot shows 84.0% of residues in the most favored regions, 15.2% of residues in allowed regions, and 0.8% disallowed residues (Table 1).

### Structure analysis and illustrations

All figures were prepared using the program Pymol^68^ and Chimera^69^. Atomic models of the full length PC-portal protein was generated by manually placing the crystal structure of PC-portal core inside the 8.7 Å asymmetric reconstruction of P22 procapsid (EMD-1828)^5^ followed by rigid body refinement in Chimera^69^. Residues 594–631 were built by hand and refined against the EM-density using refinement using *phenix.real_space_refine*^66^. This region of the pseudo-atomic model is therefore 12-fold averaged, while residues 7–593 observed crystallographically are asymmetric. The CC between EMD-1828 and PC-portal (res. 6–631) or MV-portal (res. 6–631) was calculated using *phenix.get_cc_mtz_pdb* (ref. 63). In both cases, atomic models were placed in the EM density (filtered to 14 Å resolution) by molecular replacement and rigid-body refined in Chimera^69^.

### Circular Dichroism and thermal denaturation

CD scans were acquired on a Jasco J-810 spectropolarimeter equipped with a Peltier temperature control system using a 0.1 cm quartz cuvette (Starna Cells, Inc.)^70^. Assays were carried out using purified portal-602 and portal-725 dissolved at 1 μM final concentration in 10 mM HEPES, pH 7.4 and 70 mM NaCl. CD scans were measured between 196 and 260 nm at 5 °C. Secondary structure content estimated from the measured ellipticity using the K2d method^35^.

### Data availability

The coordinates and structure factors for P22 PC-portal core and a refined model of MV-portal protein have been deposited in the Protein Data Bank, with the accession codes 5JJ1 and 5JJ3, respectively. The data that support the findings of this study are available from the corresponding author on request.

## Additional information

**How to cite this article:** Lokareddy, R. K. *et al*. Portal protein functions akin to a DNA-sensor that couples genome-packaging to icosahedral capsid maturation. *Nat. Commun.* **8,** 14310 doi: 10.1038/ncomms14310 (2017).

**Publisher's note**: Springer Nature remains neutral with regard to jurisdictional claims in published maps and institutional affiliations.

## Supplementary Material

Supplementary Information Supplementary Figures, Supplementary Table, Supplementary Acknowledgements and Supplementary References

## Figures and Tables

**Table 1:** Crystallographic data collection and refinement statistics.

	**PC-portal core** **(res. 1–602)**	**MV-portal protein** **(res. 1–725)**
*Data collection*		
Space group	P4_2_	I4
Cell dimensions		
* a*, *b*, *c* (Å)	316.8, 316.8, 138.6	409.0, 408.0, 260.0
* α*, *β*, *γ* (°)	90.0, 90.0, 90.0	90.0, 90.0, 90.0
Wavelength (Å)	1.078	0.970
Resolution (Å)	50–3.3 (3.42–3.30)	60–7.0 (7.25–7.0)
No. reflections (tot/unique)	9,911,319/206,085	345,714/33,729
*R*_sym_	30.1 (87.3)	14.6 (63.0)
*R*_pim_	10.5 (36.8)	8.4 (50.9)
*I*/σ*I*	10.7 (1.8)	19.4 (3.3)
Completeness (%)	100.0 (99.9)	98.8 (95.6)
Redundancy	8.8 (7.7)	3.9 (3.4)
		
*Refinement*		
Resolution (Å)	15–3.3	15–7.0
No. reflections	173,371	30,078
*R*_work_/*R*_free_*	29.5/31.5	23.9/25.9
No. protomers	12	12
No. protein atoms	56,578	64,536
*B*-factor (Å^2^)	70.6	118
R.m.s deviations		
Bond lengths (Å)	0.004	0.004
Bond angles (°)	1.033	1.014

Values in parentheses are for highest-resolution shells.

^*^R_free_ was calculated using ∼2,000 reflections selected in thin resolution shells.

## References

[b1] Suhanovsky M. M. & Teschke C. M. Nature's favorite building block: deciphering folding and capsid assembly of proteins with the HK97-fold. Virology 479–480, 487–497 (2015). 10.1016/j.virol.2015.02.055 PMC4424165 25864106

[b2] Cardone G., Heymann J. B., Cheng N., Trus B. L. & Steven A. C. Procapsid assembly, maturation, nuclear exit: dynamic steps in the production of infectious herpesvirions. Adv. Exp. Med. Biol. 726, 423–439 (2012). 22297525 10.1007/978-1-4614-0980-9_19 PMC3475206

[b3] Guo P. . Common mechanisms of DNA translocation motors in bacteria and viruses using one-way revolution mechanism without rotation. Biotechnol. Adv. 32, 853–872 (2014). 24913057 10.1016/j.biotechadv.2014.01.006 PMC4052234

[b4] Bhardwaj A., Olia A. S. & Cingolani G. Architecture of viral genome-delivery molecular machines. Curr. Opin. Struct. Biol. 25, 1–8 (2014). 24878339 10.1016/j.sbi.2013.10.005 PMC4040186

[b5] Chen D. H. . Structural basis for scaffolding-mediated assembly and maturation of a dsDNA virus. Proc. Natl Acad. Sci. USA 108, 1355–1360 (2011). 21220301 10.1073/pnas.1015739108 PMC3029737

[b6] Parent K. N. . P22 coat protein structures reveal a novel mechanism for capsid maturation: stability without auxiliary proteins or chemical crosslinks. Structure 18, 390–401 (2010). 20223221 10.1016/j.str.2009.12.014 PMC2951021

[b7] Lander G. C. . The structure of an infectious p22 Virion shows the signal for headful DNA packaging. Science 312, 1791–1795 (2006). 16709746 10.1126/science.1127981

[b8] Tang J. . Peering down the barrel of a bacteriophage portal: the genome packaging and release valve in p22. Structure 19, 496–502 (2011). 21439834 10.1016/j.str.2011.02.010 PMC3075339

[b9] Simpson A. A. . Structure of the bacteriophage phi29 DNA packaging motor. Nature 408, 745–750 (2000). 11130079 10.1038/35047129 PMC4151180

[b10] Guasch A. . Detailed architecture of a DNA translocating machine: the high-resolution structure of the bacteriophage phi29 connector particle. J. Mol. Biol. 315, 663–676 (2002). 11812138 10.1006/jmbi.2001.5278

[b11] Lebedev A. A. . Structural framework for DNA translocation via the viral portal protein. Embo J. 26, 1984–1994 (2007). 17363899 10.1038/sj.emboj.7601643 PMC1847669

[b12] Agirrezabala X. . Structure of the connector of bacteriophage T7 at 8A resolution: structural homologies of a basic component of a DNA translocating machinery. J. Mol. Biol. 347, 895–902 (2005). 15784250 10.1016/j.jmb.2005.02.005

[b13] Olia A. S., Prevelige P. E. Jr, Johnson J. E. & Cingolani G. Three-dimensional structure of a viral genome-delivery portal vertex. Nat. Struct. Mol. Biol. 18, 597–603 (2011). 21499245 10.1038/nsmb.2023 PMC3087855

[b14] Sun L. . Cryo-EM structure of the bacteriophage T4 portal protein assembly at near-atomic resolution. Nat. Commun. 6, 7548 (2015). 26144253 10.1038/ncomms8548 PMC4493910

[b15] Zheng H. . A conformational switch in bacteriophage p22 portal protein primes genome injection. Mol. Cell 29, 376–383 (2008). 18280242 10.1016/j.molcel.2007.11.034 PMC3936403

[b16] Trus B. L. . Structure and polymorphism of the UL6 portal protein of herpes simplex virus type 1. J. Virol. 78, 12668–12671 (2004). 15507654 10.1128/JVI.78.22.12668-12671.2004 PMC525097

[b17] Chang J., Weigele P., King J., Chiu W. & Jiang W. Cryo-EM asymmetric reconstruction of bacteriophage P22 reveals organization of its DNA packaging and infecting machinery. Structure 14, 1073–1082 (2006). 16730179 10.1016/j.str.2006.05.007

[b18] Liu X. . Structural changes in a marine podovirus associated with release of its genome into Prochlorococcus. Nat. Struct. Mol. Biol. 17, 830–836 (2010). 20543830 10.1038/nsmb.1823 PMC2924429

[b19] Jiang W. . Structure of epsilon15 bacteriophage reveals genome organization and DNA packaging/injection apparatus. Nature 439, 612–616 (2006). 16452981 10.1038/nature04487 PMC1559657

[b20] Rao V. B. & Feiss M. The bacteriophage DNA packaging motor. Annu. Rev. Genet. 42, 647–681 (2008). 18687036 10.1146/annurev.genet.42.110807.091545

[b21] Casjens S. R. The DNA-packaging nanomotor of tailed bacteriophages. Nat. Rev. Microbiol. 9, 647–657 (2011). 21836625 10.1038/nrmicro2632

[b22] Roy A. & Cingolani G. Structure of p22 headful packaging nuclease. J. Biol. Chem. 287, 28196–28205 (2012). 22715098 10.1074/jbc.M112.349894 PMC3431676

[b23] Roy A., Bhardwaj A., Datta P., Lander G. C. & Cingolani G. Small terminase couples viral DNA binding to genome-packaging ATPase activity. Structure 20, 1403–1413 (2012). 22771211 10.1016/j.str.2012.05.014 PMC3563279

[b24] McNulty R. . Architecture of the complex formed by large and small terminase subunits from bacteriophage P22. J. Mol. Biol. 427, 3285–3299 (2015). 26301600 10.1016/j.jmb.2015.08.013 PMC4587339

[b25] Casjens S. & Weigele P. in *Viral Genome Packaging Machines: Genetics, Structure and Mechanism* (ed Catalano, C.), 80–88 (Landes Publishing, 2005).

[b26] Padilla-Meier G. P. & Teschke C. M. Conformational changes in bacteriophage P22 scaffolding protein induced by interaction with coat protein. J. Mol. Biol. 410, 226–240 (2011). 21605566 10.1016/j.jmb.2011.05.006 PMC3125579

[b27] Olia A. S. . Binding-induced stabilization and assembly of the phage P22 tail accessory factor gp4. J. Mol. Biol. 363, 558–576 (2006). 16970964 10.1016/j.jmb.2006.08.014

[b28] Olia A. S., Bhardwaj A., Joss L., Casjens S. & Cingolani G. Role of gene 10 protein in the hierarchical assembly of the bacteriophage P22 portal vertex structure. Biochemistry 46, 8776–8784 (2007). 17620013 10.1021/bi700186e

[b29] Andrews D. . Bacteriophage P22 tail accessory factor GP26 is a long triple-stranded coiled-coil. J. Biol. Chem. 280, 5929–5933 (2005). 15591072 10.1074/jbc.C400513200

[b30] Olia A. S., Casjens S. & Cingolani G. Structure of phage P22 cell envelope-penetrating needle. Nat. Struct. Mol. Biol. 14, 1221–1226 (2007). 18059287 10.1038/nsmb1317

[b31] Cingolani G., Moore S. D., Prevelige P. E. Jr & Johnson J. E. Preliminary crystallographic analysis of the bacteriophage P22 portal protein. J. Struct. Biol. 139, 46–54 (2002). 12372319 10.1016/s1047-8477(02)00512-9

[b32] Lorenzen K., Olia A. S., Uetrecht C., Cingolani G. & Heck A. J. Determination of stoichiometry and conformational changes in the first step of the P22 tail assembly. J. Mol. Biol. 379, 385–396 (2008). 18448123 10.1016/j.jmb.2008.02.017 PMC2768609

[b33] Casjens S. . Bacteriophage P22 portal protein is part of the gauge that regulates packing density of intravirion DNA. J. Mol. Biol. 224, 1055–1074 (1992). 1569567 10.1016/0022-2836(92)90469-z

[b34] Kang S., Poliakov A., Sexton J., Renfrow M. B. & Prevelige P. E. Jr Probing conserved helical modules of portal complexes by mass spectrometry-based hydrogen/deuterium exchange. J. Mol. Biol. 381, 772–784 (2008). 18621389 10.1016/j.jmb.2008.03.004 PMC2593103

[b35] Whitmore L. & Wallace B. A. DICHROWEB, an online server for protein secondary structure analyses from circular dichroism spectroscopic data. Nucleic Acids Res. 32, W668–W673 (2004). 15215473 10.1093/nar/gkh371 PMC441509

[b36] Tang L., Marion W. R., Cingolani G., Prevelige P. E. & Johnson J. E. Three-dimensional structure of the bacteriophage P22 tail machine. Embo J. 24, 2087–2095 (2005). 15933718 10.1038/sj.emboj.7600695 PMC1150889

[b37] Sun S. . The structure of the phage T4 DNA packaging motor suggests a mechanism dependent on electrostatic forces. Cell 135, 1251–1262 (2008). 19109896 10.1016/j.cell.2008.11.015 PMC12969755

[b38] Lander G. C. . The P22 tail machine at subnanometer resolution reveals the architecture of an infection conduit. Structure 17, 789–799 (2009). 19523897 10.1016/j.str.2009.04.006 PMC2714705

[b39] Dixit A. B., Ray K., Thomas J. A. & Black L. W. The C-terminal domain of the bacteriophage T4 terminase docks on the prohead portal clip region during DNA packaging. Virology 446, 293–302 (2013). 24074593 10.1016/j.virol.2013.07.011 PMC3903156

[b40] Dauden M. I. . Large terminase conformational change induced by connector binding in bacteriophage T7. J. Biol. Chem. 288, 16998–17007 (2013). 23632014 10.1074/jbc.M112.448951 PMC3675631

[b41] Morita M., Tasaka M. & Fujisawa H. Structural and functional domains of the large subunit of the bacteriophage T3 DNA packaging enzyme: importance of the C-terminal region in prohead binding. J. Mol. Biol. 245, 635–644 (1995). 7844832 10.1006/jmbi.1994.0052

[b42] Yeo A. & Feiss M. Specific interaction of terminase, the DNA packaging enzyme of bacteriophage lambda, with the portal protein of the prohead. J. Mol. Biol. 245, 141–150 (1995). 7799432 10.1006/jmbi.1994.0013

[b43] Mao H. . Structural and molecular basis for coordination in a viral DNA packaging motor. Cell Rep. 14, 2017–2029 (2016). 26904950 10.1016/j.celrep.2016.01.058 PMC4824181

[b44] Agirrezabala X. . Maturation of phage T7 involves structural modification of both shell and inner core components. EMBO J. 24, 3820–3829 (2005). 16211007 10.1038/sj.emboj.7600840 PMC1276722

[b45] Liu S. . A viral packaging motor varies its DNA rotation and step size to preserve subunit coordination as the capsid fills. Cell 157, 702–713 (2015). 10.1016/j.cell.2014.02.034 PMC4003460 24766813

[b46] Al-Zahrani A. S. . The small terminase, gp16, of bacteriophage T4 is a regulator of the DNA packaging motor. J. Biol. Chem. 284, 24490–24500 (2009). 19561086 10.1074/jbc.M109.025007 PMC2782041

[b47] Zhao H., Christensen T. E., Kamau Y. N. & Tang L. Structures of the phage Sf6 large terminase provide new insights into DNA translocation and cleavage. Proc. Natl Acad. Sci. USA 110, 8075–8080 (2013). 23630261 10.1073/pnas.1301133110 PMC3657791

[b48] Oliveira L., Cuervo A. & Tavares P. Direct interaction of the bacteriophage SPP1 packaging ATPase with the portal protein. J. Biol. Chem. 285, 7366–7373 (2010). 20056615 10.1074/jbc.M109.061010 PMC2844185

[b49] Hegde S., Padilla-Sanchez V., Draper B. & Rao V. B. Portal-large terminase interactions of the bacteriophage T4 DNA packaging machine implicate a molecular lever mechanism for coupling ATPase to DNA translocation. J. Virol. 86, 4046–4057 (2012). 22345478 10.1128/JVI.07197-11 PMC3318623

[b50] Hilbert B. J. . Structure and mechanism of the ATPase that powers viral genome packaging. Proc. Natl Acad. Sci. USA 112, E3792–E3799 (2015). 26150523 10.1073/pnas.1506951112 PMC4517215

[b51] Cingolani G. & Duncan T. M. Structure of the ATP synthase catalytic complex (F(1)) from *Escherichia coli* in an autoinhibited conformation. Nat. Struct. Mol. Biol. 18, 701–707 (2011). 21602818 10.1038/nsmb.2058 PMC3109198

[b52] Harvey S. C. The scrunchworm hypothesis: transitions between A-DNA and B-DNA provide the driving force for genome packaging in double-stranded DNA bacteriophages. J. Struct. Biol. 189, 1–8 (2016). 10.1016/j.jsb.2014.11.012 PMC4357361 25486612

[b53] Fang H., Jing P., Haque F. & Guo P. Role of channel lysines and the ‘push through a one-way valve' mechanism of the viral DNA packaging motor. Biophys. J. 102, 127–135 (2012). 22225806 10.1016/j.bpj.2011.11.4013 PMC3250684

[b54] Grimes S., Ma S., Gao J., Atz R. & Jardine P. J. Role of phi29 connector channel loops in late-stage DNA packaging. J. Mol. Biol. 410, 50–59 (2011). 21570409 10.1016/j.jmb.2011.04.070 PMC3140409

[b55] Baumann R. G., Mullaney J. & Black L. W. Portal fusion protein constraints on function in DNA packaging of bacteriophage T4. Mol. Microbiol. 61, 16–32 (2006). 16824092 10.1111/j.1365-2958.2006.05203.x

[b56] Hugel T. . Experimental test of connector rotation during DNA packaging into bacteriophage phi29 capsids. PLoS Biol. 5, e59 (2007). 17311473 10.1371/journal.pbio.0050059 PMC1800307

[b57] Bhardwaj A., Olia A. S., Walker-Kopp N. & Cingolani G. Domain organization and polarity of tail needle GP26 in the portal vertex structure of bacteriophage P22. J. Mol. Biol. 371, 374–387 (2007). 17574574 10.1016/j.jmb.2007.05.051

[b58] Cortines J. R., Motwani T., Vyas A. A. & Teschke C. M. Highly specific salt bridges govern bacteriophage P22 icosahedral capsid assembly: identification of the site in coat protein responsible for interaction with scaffolding protein. J. Virol. 88, 5287–5297 (2014). 24600011 10.1128/JVI.00036-14 PMC4019102

[b59] D'Lima N. G. & Teschke C. M. A molecular staple: D-Loops in the I domain of bacteriophage P22 coat protein make important intercapsomer contacts required for procapsid assembly. J. Virol. 89, 10569–10579 (2015). 26269173 10.1128/JVI.01629-15 PMC4580156

[b60] Mastronarde D. N. Automated electron microscope tomography using robust prediction of specimen movements. J. Struct. Biol. 152, 36–51 (2005). 16182563 10.1016/j.jsb.2005.07.007

[b61] Wang Z. . An atomic model of brome mosaic virus using direct electron detection and real-space optimization. Nat. Commun. 5, 4808 (2014). 25185801 10.1038/ncomms5808 PMC4155512

[b62] Otwinowski Z. & Minor W. Processing of X-ray diffraction data collected in oscillation mode. Methods in Enzymology 276, 307–326 (1997). 10.1016/S0076-6879(97)76066-X 27754618

[b63] Adams P. D. . PHENIX: a comprehensive Python-based system for macromolecular structure solution. Acta Crystallogr. D Biol. Crystallogr. 66, 213–221 (2010). 20124702 10.1107/S0907444909052925 PMC2815670

[b64] McCoy A. J. . Phaser crystallographic software. J. Appl. Cryst. 40, 658–674 (2007). 19461840 10.1107/S0021889807021206 PMC2483472

[b65] Emsley P. & Cowtan K. Coot: model-building tools for molecular graphics. Acta Crystallogr. D Biol. Crystallogr. 60, 2126–2132 (2004). 15572765 10.1107/S0907444904019158

[b66] Afonine P. V., Headd J. J., Terwilliger T. C. & Adams P. D. New Tool: phenix.real_space_refine. Computational Crystallography Newsletter 4, 43–44 (2013).

[b67] Lasker K., Topf M., Sali A. & Wolfson H. J. Inferential optimization for simultaneous fitting of multiple components into a CryoEM map of their assembly. J. Mol. Biol. 388, 180–194 (2009). 19233204 10.1016/j.jmb.2009.02.031 PMC2680734

[b68] DeLano W. L. The PyMOL Molecular Graphics System, Version 1.8 Schrödinger, LLC (2002).

[b69] Pettersen E. F. . UCSF Chimera–a visualization system for exploratory research and analysis. J. Comput. Chem. 25, 1605–1612 (2004). 15264254 10.1002/jcc.20084

[b70] Bhardwaj A., Walker-Kopp N., Wilkens S. & Cingolani G. Foldon-guided self-assembly of ultra-stable protein fibers. Protein Sci. 17, 1475–1485 (2008). 18535304 10.1110/ps.036111.108 PMC2525528

