# Supplementary material for: Portal protein functions akin to a DNA-sensor that couples genome-packaging to icosahedral capsid maturation
Source: Nat Commun. 2017 Jan 30;8:14310. doi: 10.1038/ncomms14310 (PMC5290284; doi:10.1038/ncomms14310)
Supplement: Supplementary Information — Supplementary Figures, Supplementary Table, Supplementary Acknowledgements and Supplementary References [file ncomms14310-s1.pdf]

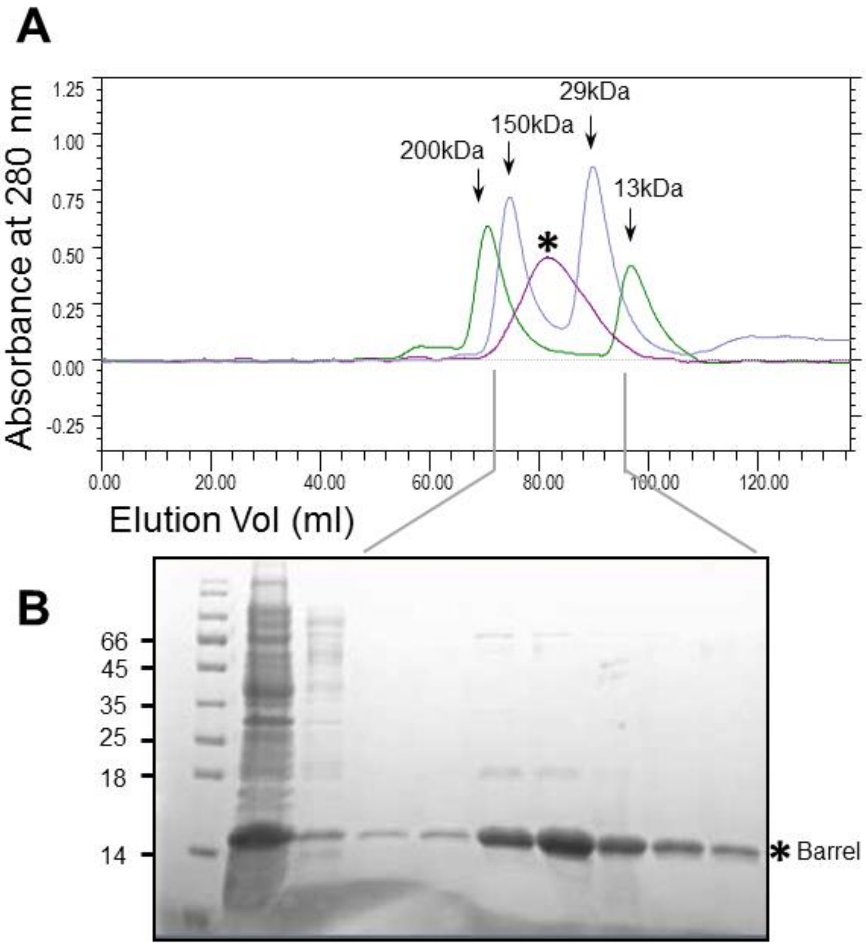

Supplementary Figure 1. The isolated barrel domain (res 603-725) does not form a dodecamer in solution. (**A**) Size exclusion chromatography analysis of the isolated barrel domain (M.W. ~14 kDa) analyzed on a Superdex 200 16/60 column (GE Healthcare) equilibrated in GF-buffer (20 mM Tris-Cl pH 8.0, 150 mM NaCl, 1 mM MgCl2, 1 mM β-mercaptoethanol). The column was calibrated using cytochrome C (12.4 kDa, elution volume = 96.9 ml), carbonic anhydrase (29 kDa, elution volume = 89.4 ml), albumin (66 kDa, elution volume = 79.8 ml), alcohol dehydrogenase (150 kDa, elution volume = 74.4 ml), β-Amylase (200 kDa, elution volume = 70.6 ml) and blue dextran (2,000 kDa) from the Gel Filtration Molecular Weight Markers Kit (Sigma). Based on calibration markers, the barrel domain has an estimated M.W. ~55 kDa, possibly consistent with a very elongated monomer or a dimer. (**B**) Coomassie blue stained SDS-PAGE of the eluted barrel domain.

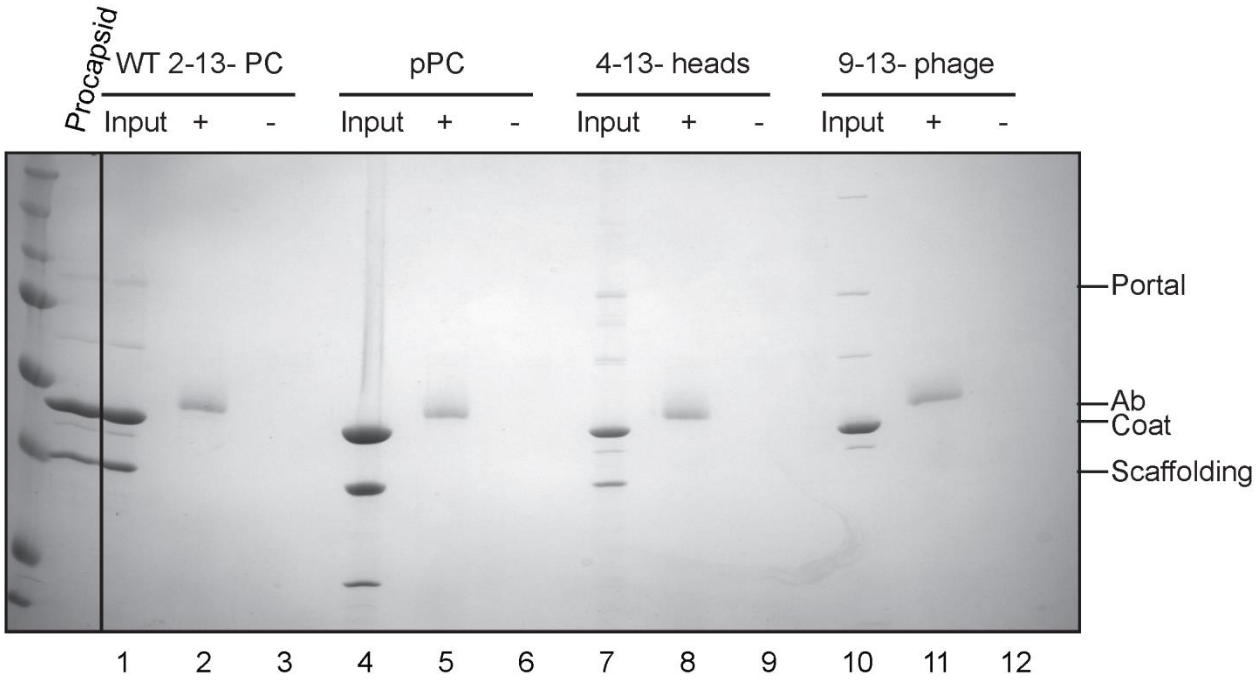

Supplementary Figure 2. Portal protein immunoprecipitation assay. Coomassie blue stained SDS-PAGE of a representative experiment. 'Input': 10 μg of control samples (see Methods); '+': proteins immunoprecipitated by the anti-stalk antibody; '-': proteins incubated with the Protein A agarose beads but without the antibody. 'WT 2^-13^- PC' are genuine P22 procapsids; 'pPC' indicates procapsids assembled *in vivo* without portal; '4^-13^- heads' are empty mature P22 heads; '9^-13^- phages' are tail-less P22 phages. The migration of portal protein, antibody IgG band (Ab), coat protein, and scaffolding protein is indicated on the right.

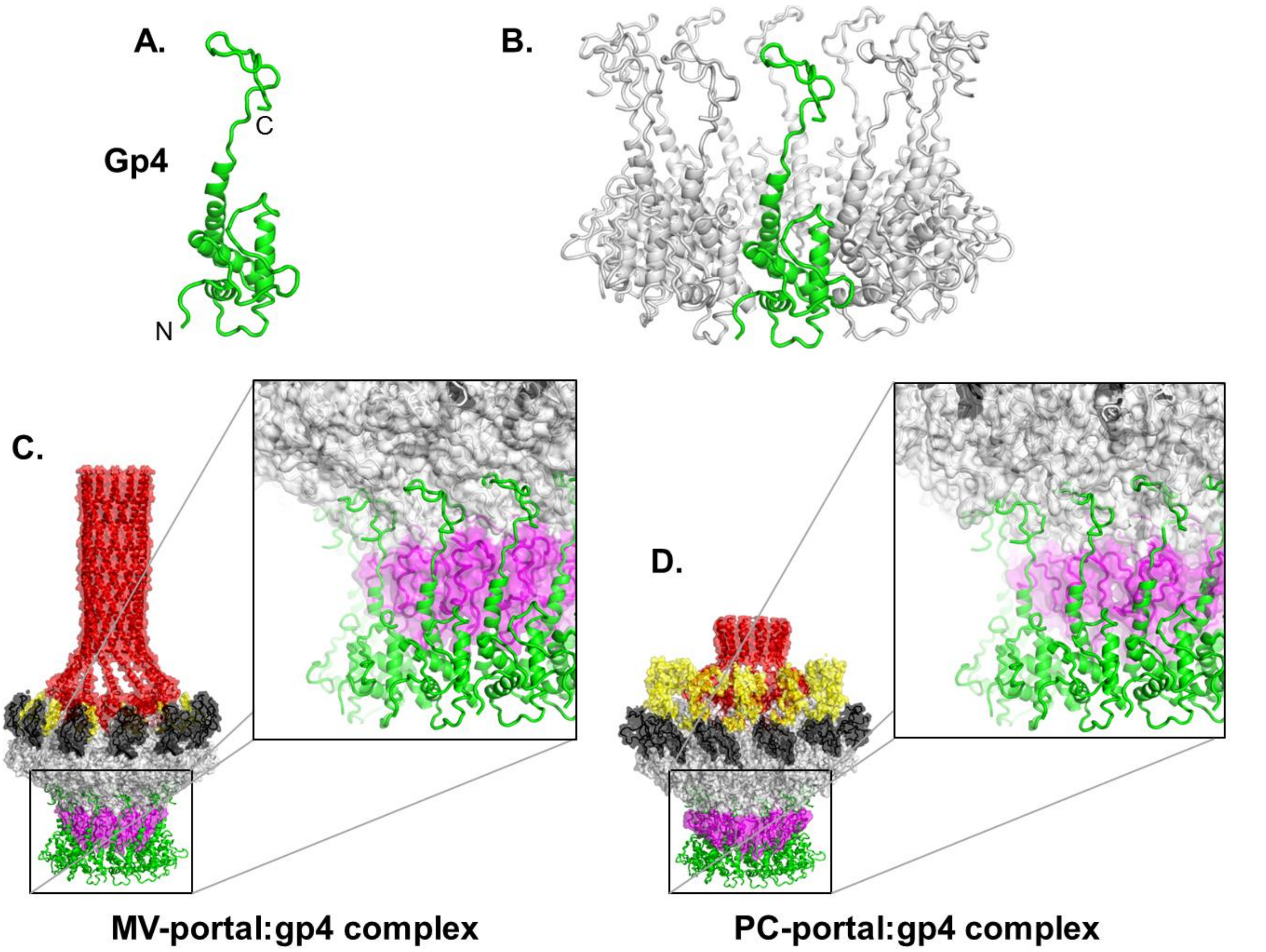

Supplementary Figure 3. Gp4 can bind both PC- and MV-portal protein. Ribbon diagram of (**A**) monomeric and (**B**) dodecameric gp4 (from pdb 3LJ4). Model of (**C**) MV-portal and (**D**) PC-portal protein bound to 12 copies of gp4. In both panels, the zoom-in window shows gp4 lateral binding at the portal protomer:protomer interface.

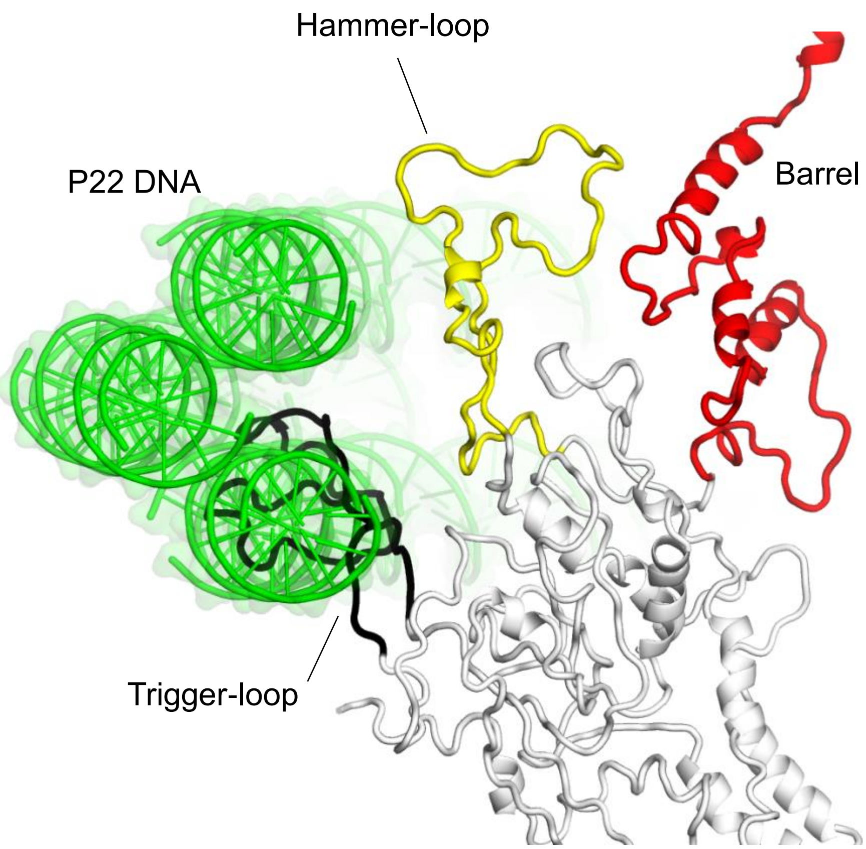

Supplementary Figure 4. Molecular model of a PC-portal protomer surrounded by the three rings of DNA visible in the cryo-EM reconstruction of the P22 mature virion [3,4]. The conformation of the trigger-loop in PC-portal protein is incompatible with packaged DNA filling the capsid.

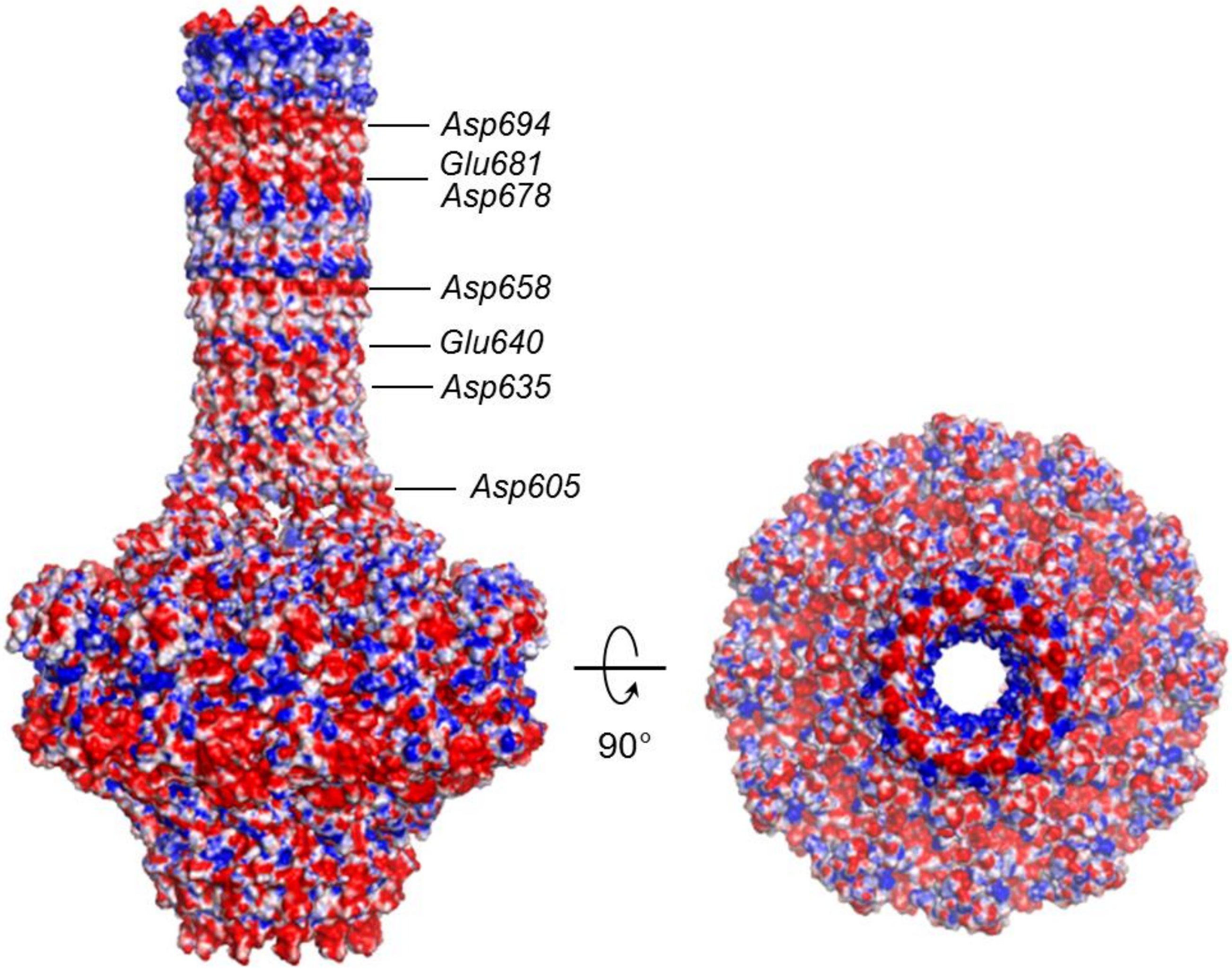

Supplementary Figure 5. The electrostatic surface charge distribution of MV-portal protein. Surface-exposed acidic residues in the barrel domain are shown by arrows. Overall, the barrel is mainly acidic between residues 605-694. Non-linear Poisson-Boltzmann electrostatic calculations were performed using APBS Tools [5] and surfaces rendered in program Pymol [6].

	PC-portal refined without NCS	PC-portal refined with NCS-restraints *	PC-portal refined with NCS-constraints
**R_{work}/R_{free} (%)**	29.5 / 31.5	30.5 / 32.7	49.4 / 50.2
**RMSD** bonds / angles (Å)	0.004 / 1.033	0.010 / 2.219	0.029 / 3.047
**Ramachandran (%)** favored / allowed / outliers	76.89/ 21.71 / 1.40	73.14 / 19.97 / 6.89	59.19 / 29.09 / 11.72
**Romater outliers (%)**	0.0	2.2	10.4
**C-beta outliers**	0	86	130
**Clashscore** **	18.9	27.3	63.1
**MolProbity Score** **	2.55	3.01	3.96

* ~42,239 torsion-angle NCS restraints generated from the 12 subunits using a *max_RMSD* cut-off of 5.5 Å, as implemented in *phenix.simple_ncs_from_pdb* [1]
** As defined in reference [2]

Supplementary Table 1. Effect of NCS on the crystallographic refinement of PC-portal protein. The final model was subjected to three macro-cycles of crystallographic refinement using *phenix.refine* [1] without imposing NCS (left column), with torsion-angle NCS restraints (central column) and with 'strict' NCS (e.g. NCS-constraints) that assume all 12 subunits are identical (right column). Each macro-cycle includes cycles of bulk solvent correction, real space, XYZ positional and individual B-factor refinement.

Supplementary Acknowledgements. We are grateful for beamtime and assistance in data collection to staff at NSLS X6A, X29 stations; SSRL 14-1 station and APS LS-CAT 21-ID-G/F stations. We wish to thank Dr. Michael Spilman (Direct Electron), Dr. Steven Ludtke (Baylor), Dr. Jaap Brink (JEOL), Dr. David Mastronarde (Boulder), and everyone at the Center for Advanced Microscopy (Michigan State) for help developing the cryo-EM setup at MSU.

Supplementary References

1. Adams, P.D. et al. PHENIX: a comprehensive Python-based system for macromolecular structure solution. *Acta Crystallogr D Biol Crystallogr* **66**, 213-21 (2010).
2. Davis, I.W. et al. MolProbity: all-atom contacts and structure validation for proteins and nucleic acids. *Nucleic Acids Res* **35**, W375-83 (2007).
3. Lander, G.C. et al. The Structure of an Infectious p22 Virion Shows the Signal for Headful DNA Packaging. *Science* (2006).
4. Tang, J. et al. Peering down the barrel of a bacteriophage portal: the genome packaging and release valve in p22. *Structure* **19**, 496-502 (2011).
5. Dolinsky, T.J., Nielsen, J.E., McCammon, J.A. & Baker, N.A. PDB2PQR: an automated pipeline for the setup of Poisson-Boltzmann electrostatics calculations. *Nucleic Acids Res* **32**, W665-7 (2004).
6. DeLano, W.L. The PyMOL Molecular Graphics System, Version 1.8 Schrödinger, LLC. (2002).
